# Influencing Factors of Subjective Cognitive Impairment in Middle-Aged and Older Adults

**DOI:** 10.3390/ijerph182111488

**Published:** 2021-10-31

**Authors:** Min Roh, Hyunju Dan, Oksoo Kim

**Affiliations:** College of Nursing, Ewha Womans University, Seoul 03760, Korea; nmlhj@hanmail.net (M.R.); hidan@hanmail.net (H.D.)

**Keywords:** subjective cognitive impairment, depressive symptoms, middle-aged, older adults, stress

## Abstract

The purpose of this study was to identify the factors affecting subjective cognitive impairment. We analyzed data from the 2019 Korea Community Health Survey and enrolled 68,546 middle-aged adults, aged 50 to 64 years, and 74,547 older adults, aged 65 years and older, in this study. Multiple logistic regression analysis was performed to identify factors influencing subjective cognitive impairment. Of the participants, 11,926 (17.4%) middle-aged and 21,880 (29.4%) older adults living in the community reported subjective cognitive impairment. Major factors that influenced subjective cognitive impairment in both middle-aged and older adults were gender, subjective stress, depressive symptoms, and alcohol drinking. In contrast to middle-aged adults, the marital status of older adults affected subjective cognitive impairment. Therefore, the factors affecting subjective cognitive impairment in middle-aged and older adults need to be considered for screening and management to prevent cognitive impairment and dementia. In particular, it is necessary to evaluate and manage stress and depressive symptoms from middle age to prevent subjective cognitive impairment.

## 1. Introduction

As the middle-aged and elderly population increases due to increased lifespans of the population, cognitive impairment related to dementia is emerging as a major health problem [1]. Aggravation of cognitive impairment eventually leads to dementia and increased medical costs [2,3]. Therefore, it is necessary to pay attention to cognitive impairment in middle age and old age.

The prevalence of mild cognitive impairment among Korean older adults over 65 years old is 22.6% [4]. Mild cognitive impairment is a preclinical stage of dementia that indicates a cognitive change between normal aging and dementia and is associated with high risk of dementia [5,6]. Cognitive impairment in old age is recoverable by 10–15% when diagnosed early [7]. Cognitive impairment decreases the quality of life of the individual as well as of the family caring for them [8]. Because subjective cognitive impairment may be a symptomatic indicator of Alzheimer’s disease [9] early screening and continuous management of subjective cognitive impairment are needed.

For screening of subjective cognitive impairment, which indicates how an individual feels and evaluates changes in overall cognitive functions such as memory, concentration, and language ability, is important [10]. The Centers for Disease Control and Prevention (CDC) in the United States investigated the subjective cognitive impairment of middle-aged people over 45 years of age as well as older adults. During 2015–2017, 11.7% of older adults aged 65 years and older and 10.8% of middle-aged people aged 45 to 64 years experienced subjective cognitive impairment [11]. Since 2018, the Korea Disease Control and Prevention Agency (KDCA) has investigated the subjective cognitive impairment of middle-aged people aged 50 and over as a new health indicator. Therefore, subjective cognitive impairment is considered one of the important health problems that needs to be managed from middle age.

According to the results of previous studies, age, education level, and degree of depression had an effect on mild cognitive impairment [12] and subjective cognitive decline in older adults [13,14]. In addition, activity ability, diabetes, smoking, exercise, subjective health status, and social relationships affected cognitive decline [15,16,17]. Legdeur et al. [18] found that smoking, high LDL, and depression were risk factors for cognitive decline in participants aged 50 to 70 years, whereas stroke and hypertension were risk factors in participants aged 70 years and older, indicating a difference by age.

Most studies on factors affecting subjective cognitive impairment have targeted older adults, and there is little research that includes the middle-aged. Identifying the factors influencing the experiences of subjective cognitive impairment in middle-aged and older adults is important for early screening and prevention of cognitive impairment. Therefore, this study aims to identify the experiences of subjective cognitive impairment in middle-aged and older adults and to identify factors affecting the experiences of subjective cognitive impairment.

## 2. Materials and Methods

### 2.1. Data Source and Samples

Data from the 2019 Korea Community Health Survey (KCHS), a nationwide cross-sectional survey, conducted by the KDCA were analyzed. In the KCHS, a stratified two-stage sampling method based on resident registration information was used. Sample residential areas were selected using a stratified sampling method, and sample households were selected using a systematic sampling method. All members aged 19 years and over in the sample households were selected as the target population. Trained investigators visited selected sample households and collected data using face-to-face interviews from August to October of 2019. In this study, of 229,099 participants in the 2019 KCHS, 68,546 middle-aged adults aged 50 to 64 years and 74,547 older adults aged 65 years and older were included in the final analysis.

### 2.2. Instruments

#### 2.2.1. Subjective Cognitive Impairment

Subjective cognitive impairment was measured using the Behavioral Risk Factor Surveillance System (BRFSS) questionnaire [19]. To identify subjective cognitive impairment, participants were asked whether they had experienced more frequent or more severe mental confusion or memory loss in the past year. Participants answered “yes” or “no” depending on whether they subjective cognitive impairment.

#### 2.2.2. Physical Activity

Physical activity was measured using the Korean version of the International Physical Activity Questionnaire Short Form. The test-retest reliability and criterion validity of the tool were confirmed [20]. Participants were asked how many days they performed vigorous physical activity, moderate physical activity, and walking for at least 10 min over the past week. Vigorous physical activity includes activities that are very strenuous or require a lot of breathlessness such as running, climbing, biking at a high speed, swimming fast, and carrying heavy objects. Moderate physical activity includes activities that are a bit strenuous or that result in some shortness of breath, such as slow swimming, badminton, table tennis, and carrying light objects.

#### 2.2.3. Subjective Stress

Subjective stress was measured as that felt during normal daily life (work, housework, etc.). The levels of stress were ‘very much,’ ‘a lot,’ ‘a little,’ and ‘hardly any.’

#### 2.2.4. Depressive Symptoms

Depressive symptoms were measured using the Patient Health Questionnaire-9 (PHQ-9) [21]. The PHQ-9 measures nine factors of displeasure, fatigue, appetite change, guilt, worthlessness, decreased concentration, slow movement, restlessness, and suicidal ideation. Each question was measured on a 4-point Likert scale, with total scores ranging from 0 to 27. The depressive symptoms were classified into five levels: minimal (0–4), mild (5–9), moderate (10–14), moderately severe (15–19), and severe (20–27). In this study, the Cronbach’s alpha of the PHQ-9 was 0.803.

#### 2.2.5. Social Network

Participants were asked about the frequency of contact with relatives including family members, neighbors, and friends but excluding relatives or family members living with them. Contact included only face-to-face interactions or phone calls. The frequency of contact was classified as none, 1 to 3 times per month, 1 to 3 times per week, and 4 or more times per week.

### 2.3. Ethical Considerations

We obtained approval from the KDCA to use the 2019 KCHS data. In addition, we received an exemption from deliberation for the 2019 KCHS data analysis from the institutional review board of the university (IRB No. ewha-202105-0005-01). The KCHS data are available in the public domain and do not include personal information. The KCHS was conducted after obtaining written consent from the participants.

### 2.4. Data Analysis

Data analysis was performed using SPSS version 26 (IBM, Armonk, NY, USA). Frequency, percentage, mean, and standard deviation were calculated to confirm the general and health-related characteristics, social support, and psychosocial characteristics of the participants. The differences of demographic and health-related characteristics between middle-aged and older adults were analyzed using chi-square tests and *t*-tests. Multiple logistic regression analyses were performed to identify factors influencing subjective cognitive impairment.

## 3. Results

The mean age of the participants was 56.6 years among the middle-aged and 73.9 years among the older adults. Of the 68,527 middle-aged participants, 11,926 (17.4%) reported subjective cognitive impairment; 21,880 (29.4%) of 74,471 older adults reported subjective cognitive impairment (χ^2^ = 2878.42, *p* < 0.001) (Table 1).

Smokers comprised 42.9% of middle-aged and 37.7% of older adults (χ^2^ = 393.04, *p* < 0.001), and alcohol drinkers comprised 86.5% of middle-aged and 69.3% of older adults (χ^2^ = 6219.73, *p* < 0.001). Of middle-aged adults, 27.1% had hypertension and 11.3% had diabetes; of older adults, 54.5% had hypertension and 22.4% had diabetes (χ^2^ = 10,924.53, *p* < 0.001; χ^2^ = 3131.99, *p* < 0.001). Minimal depressive symptoms were reported by 87.6% of middle-aged and 80.2% of older adults (χ^2^ = 1503.47, *p* < 0.001) (Table 2).

Table 3 shows the factors influencing subjective cognitive impairment in the middle-aged group. The older were the participants, the more frequent was the subjective cognitive impairment they experienced (odds ratio [OR] = 1.04, 95% confidence interval [CI]: 1.03–1.04). Women were more likely to experience subjective cognitive impairment than men (OR = 1.59, 95% CI: 1.46–1.73), and participants with a monthly income less than $1000 were more likely to experience subjective cognitive impairment than were those whose monthly income was more than $5000 (OR = 1.13, 95% CI: 1.014–1.274). Alcohol drinkers were more likely to experience subjective cognitive impairment than non-drinkers (OR = 1.35, 95% CI: 1.25–1.46). Participants who performed more frequent vigorous physical activity were more likely to experience subjective cognitive impairment (OR = 1.02, 95% CI: 1.01–1.04). Participants who had no contact with family and relatives for one month were more likely to experience subjective cognitive impairment than were those who had contact more than four times a week (OR = 1.11, 95% CI: 1.01–1.22). Participants with too much stress were more likely to experience subjective cognitive impairment than were participants with hardly any stress (OR = 1.96, 95% CI: 1.65–2.34). Participants with minimal depressive symptoms were more likely to experience subjective cognitive impairment than participants with moderately severe (OR = 4.85, 95% CI: 3.64–6.46) or severe depressive symptoms (OR = 7.58, 95% CI: 5.05–11.38).

Table 4 shows the factors influencing subjective cognitive impairment in older adults. Women were more likely to experience subjective cognitive impairment than men (OR = 1.34, 95% CI: 1.24–1.44). Compared to married participants, participants who answered single/other (OR = 1.14, 95% CI: 1.03–1.25) were more likely to experience subjective cognitive impairment. Those living with a spouse were more likely to experience subjective cognitive impairment than were those living alone (OR = 1.16, 95% CI: 1.04–1.29). Participants with elementary or lower education level were more likely to experience subjective cognitive impairment than were those with college or higher education (OR = 1.16, 95% CI: 1.06–1.27). Smokers were more likely to experience subjective cognitive impairment than non-smokers (OR = 1.09, 95% CI: 1.01–1.18), and alcohol drinkers were more likely to experience subjective cognitive impairment than non-drinkers (OR = 1.25, 95% CI: 1.18–1.31). Participants who had no contact with family and relatives for one month (OR = 1.25, 95% CI: 1.16–1.34) and those who had contact 1–3 times per month (OR = 1.15, 95% CI: 1.07–1.22) were more likely to experience subjective cognitive impairment than were those who had contact more than four times a week. Participants with too much stress were more likely to experience subjective cognitive impairment than were participants with hardly any stress (OR = 1.75, 95% CI: 1.51–2.04). Participants with minimal depressive symptoms were more likely to experience subjective cognitive impairment than were participants with moderately severe (OR = 4.26, 95% CI: 3.46–5.24) or severe depressive symptoms (OR = 6.90, 95% CI: 5.02–9.50).

## 4. Discussion

The purpose of this study was to identify the factors affecting experiences of subjective cognitive impairment. In this study, 17.4% of middle-aged adults and 29.4% of older adults experienced subjective cognitive impairment. The incidence of cognitive impairment increases with age [22], and the risk of cognitive impairment increases in older adults with subjective cognitive impairment [23]. However, the high rate of subjective cognitive impairment in middle-aged people in this study indicates that screening tests are needed to identify subjective cognitive impairment not only in older adults, but also in middle-aged adults.

In this study, age, gender, and education level affected subjective cognitive impairment in both middle-aged and older adults. The results of this study support previous studies that reported an increased risk of subjective cognitive impairment in female participants and those with older age and lower education level [24,25]. Therefore, healthcare providers need to pay attention to and manage the subjective cognitive function changes of older women and older adults with low educational level. In this study, the lower the income of middle-aged participants was, the higher the subjective cognitive impairment risk was. This supports the results of a previous study in the United States [26] which showed that the risk of subjective cognitive impairment increased as the annual household income of participants aged 45 or older decreased. Therefore, it is necessary to conduct early screening tests for subjective cognitive impairment targeting middle-aged adults with low economic status.

Unlike in middle-aged adults, marital status and living arrangement in old age were significant factors influencing subjective cognitive impairment. In the elderly, the risk of subjective cognitive impairment in singles increased compared to that of participants with spouses. A previous study [27] reported that unmarried older adults were vulnerable to memory decline, but the relationship between marital status and subjective cognitive impairment was not reported, so further research is needed. In this study, the risk of subjective cognitive impairment was higher in older adults living with a spouse than in older adults living alone. Differences in subjective cognitive function according to household type among older adults are considered to be related to emotional attachment and interaction with family members living together. Kim [28] reported that the numbers of social activities and meetings with friends and relatives among elderly people living alone were higher than those of elderly people with a spouse. However, in this study, marital status and living arrangement showed contradictory results among factors affecting subjective cognitive impairment in older adults. Therefore, further research is needed on the relationship between marital status, living arrangement, and subjective cognitive function.

Diabetes affected subjective cognitive impairment in both middle-aged and older adult participants. In previous studies, diabetes was identified as an influencing factor of cognitive decline [16,17]. Since the duration and complications of diabetes were not investigated in this study, further research is needed to determine whether there is a difference in subjective cognitive impairment according to diabetes-related characteristics. It is known that active physical activity during mid-to-late life may contribute to prevention of subjective cognitive impairment in late-life [29], and that physical activity and exercise level delay the onset of dementia [30]. In this study, the level of physical activity had an effect on subjective cognitive impairment in both middle and old age.

Depressive symptoms and subjective stress were major risk factors influencing subjective cognitive impairment in middle-aged and older adults in this study. In previous studies, depression [31,32,33] and stress [34] affected subjective cognitive impairment, which was consistent with the results of this study. Subjective cognitive impairment and depressive symptoms are closely related, and a high degree of depressive symptoms may increase the risk of subjective cognitive decline [13,14]. Therefore, to prevent subjective cognitive impairment, it is necessary to manage depressive symptoms from middle age. Perceived stress level was reported as a predictor of subjective memory complaints [35]. Therefore, it is necessary to evaluate and manage stress and depressive symptoms from middle age to prevent subjective cognitive impairment.

This study has several limitations. Since this study investigated subjective indicators to evaluate subjective cognitive impairment, it is necessary to consider objective test results to evaluate cognitive function in future studies. In this study, the selection of tools was limited because secondary analysis was performed using the national data. Subjective cognitive impairment and subjective stress tools consist of a single item. However, in a large prospective epidemiologic study, a single item is used practically, and it is reported that the single item subjective stress tool shows reliability and validity similar to the multi-item tool [36]. Analysis of cross-sectional data limited the identification of causal relationships between variables. However, the study identified factors influencing subjective cognitive impairment in middle-aged and older adults living in the community based on Korean national data.

## 5. Conclusions

In this study, 17.4% of middle-aged and 29.4% of older adults living in the community experienced subjective cognitive impairment. Gender, subjective stress, depressive symptoms, and alcohol drinking were major factors influencing subjective cognitive impairment in both middle-aged and older adults. In contrast to middle-aged adults, the marital status of older adults had an effect on subjective cognitive impairment. It is necessary to screen the factors affecting subjective cognitive impairment according to age group, middle-aged vs. elderly, and to manage these factors to prevent cognitive impairment and dementia.

## Figures and Tables

**Table 1:** Characteristics of the participants (N = 143,093).

Variables	Middle-Aged	Older Adults	χ^2^	*p*
	M ± SE or N (%)	M ± SE or N (%)		
Sex				
Male	30,978 (49.2)	30,983 (44.6)	395.97	<0.001
Female	37,568 (50.8)	43,564 (55.4)		
Marital Status				
Married	55,386 (81.7)	47,293 (65.9)	4572.78	<0.001
Single/others	13,056 (18.3)	27,207 (34.1)		
Monthly income (USD)				
≤1000	6607 (7.0)	33,284 (36.9)	27,932.54	<0.001
1001~3000	21,767 (29.4)	22,733 (37.9)		
3001~5000	15,514 (27.0)	5633 (11.8)		
≥5000	23,023 (36.6)	11,364 (13.4)		
Education level				
Elementary School or less	9542 (8.6)	44,692 (48.1)	35,421.98	<0.001
Middle school	12,424 (14.2)	12,699 (19.4)		
High school	29,083 (44.0)	11,566 (20.7)		
College or more	17,392 (33.2)	5536 (11.8)		
Subjective cognitive impairment				
Yes	11,926 (17.4)	21,880 (29.4)	2878.42	<0.001
No	56,601 (82.6)	52,591 (70.6)		
Living arrangement				
Living alone	8105 (9.6)	19,156 (20.3)	16,361.83	<0.001
Living with spouse	26,073 (28.6)	37,690 (48.2)		
Living with parents or/and children	25,655 (48.5)	9341 (17.6)		
Others	8648 (13.3)	8323 (13.9)		
Frequency of family and relatives contact				
None	9701 (16.3)	9683 (14.9)	656.75	<0.001
1–3 times/month	18,720 (31.5)	17,150 (26.3)		
1–3 times/week	20,136 (27.3)	23,682 (30.4)		
≥4 times/week	19,969 (24.9)	23,999 (28.5)		
Frequency of friend contact				
None	11,539 (16.0)	22,329 (27.7)	4100.87	<0.001
1–3 times/month	20,561 (32.2)	15,641 (23.7)		
1–3 times/week	20,877 (31.6)	16,731 (24.1)		
≥4 times/week	15,529 (20.2)	19,690 (24.5)		
Frequency of neighbor contact				
None	18,919 (39.7)	12,058 (28.3)	5157.92	<0.001
1–3 times/month	9666 (16.5)	6578 (11.6)		
1–3 times/week	16,598 (22.1)	16,230 (21.8)		
≥4 times/week	23,065 (21.6)	39,490 (38.2)		

**Table 2:** Health related characteristics of the participants (N = 143,093).

Variables	Middle-Aged	Older Adults	χ^2^ or *t*	*p*
	M ± SE or N (%)	M ± SE or N (%)		
Smoking				
Yes	27,369 (42.9)	26,312 (37.7)	393.04	<0.001
No	41,172 (57.1)	48,230 (62.3)		
Alcohol drinking				
Yes	57,052 (86.5)	48,912 (69.3)	6219.73	<0.001
No	11,490 (13.5)	25,632 (30.7)		
Sleep (hours/day)	6.45 ± 0.01	6.47 ± 0.01	2.44	0.015
Vigorous physical activity (days/week)	0.88 ± 0.01	0.48 ± 0.01	−32.61	<0.001
Moderate physical activity (days/week)	1.49 ± 0.01	1.15 ± 0.01	−21.46	<0.001
Walking (days/week)	4.17 ± 0.01	4.08 ± 0.01	−4.72	<0.001
Hypertension				
Yes	19,775 (27.1)	40,947 (54.5)	10,924.53	<0.001
No	48,766 (72.9)	33,563 (45.2)		
Diabetes				
Yes	8426 (11.3)	16,069 (22.4)	3131.99	<0.001
No	60,114 (88.7)	58,453 (77.6)		
Subjective stress				
Very much	1614 (2.5)	1662 (2.3)	5410.11	<0.001
A lot	12,323 (19.2)	10,713 (14.5)		
A little	39,382 (58.4)	32,673 (45.7)		
Hardly	15,223 (19.9)	29,383 (37.5)		
Depressive symptoms				
Minimal (0–4)	60,304 (87.6)	59,639 (80.2)	1503.47	<0.001
Mild (5–9)	6494 (9.9)	10,777 (14.8)		
Moderate (10–14)	1069 (1.7)	2308 (3.3)		
Moderately severe (15–19)	350 (0.5)	806 (1.2)		
Severe (20–27)	168 (0.3)	371 (0.5)		

**Table 3:** Factors influencing subjective cognitive impairment among middle-aged adults.

Variables	Model 1		Model 2		Model 3	
	OR	95% CI	OR	95% CI	OR	95% CI
Age	1.03 **	1.02–1.04	1.03 **	1.02–1.04	1.04 ***	1.03–1.04
Sex						
Male	Ref.		Ref.		Ref.	
Female	1.47 **	1.39–1.55	1.84 **	1.69–2.00	1.59 ***	1.46–1.73
Marital status						
Married	Ref.		Ref.		Ref.	
Single/others	1.16 **	1.15–1.29	1.13 *	1.02–1.26	1.03	0.92–1.15
Monthly income (USD)						
>5000	Ref.		Ref.		Ref.	
3001~5000	1.01	0.94–1.09	1.01	0.94–1.10	1.02	0.95–1.11
1001~3000	1.06	0.98–1.14	1.06	0.99–1.15	1.02	0.94–1.10
≤1000	1.50 ***	1.35–1.67	1.49 ***	1.34–1.66	1.14 *	1.01–1.27
Education level						
College or more	Ref.		Ref.		Ref.	
High school	0.96	0.89–1.03	0.94	0.88–1.01	0.930 *	0.86–1.00
Middle school	1.11 *	1.02–1.21	1.08	0.99–1.18	1.03	0.94–1.13
Elementary School or less	1.27 **	1.15–1.39	1.22 **	1.10–1.34	1.10	1.00–1.22
Living arrangement						
Living alone	Ref.		Ref.		Ref.	
Living with spouse	0.99	0.88–1.13	1.01	0.89–1.15	1.02	0.90–1.16
Living with parents or/and children	1.09	0.97–1.24	1.11	0.98–1.26	1.09	1.00–1.24
Others	1.01	0.91–1.12	1.03	0.93–1.14	0.98	0.88–1.10
Smoking						
No			Ref.		Ref.	
Yes			1.23 **	1.12–1.34	1.07	0.98–1.17
Alcohol drinking						
No			Ref.		Ref.	
Yes			1.37 ***	1.27–1.48	1.35 ***	1.25–1.46
Sleep (hours/day)			0.90 ***	0.88–0.93	0.980	1.00
Vigorous physical activity (days/week)			1.02	1.00–1.03	1.02 **	1.01–1.04
Moderate physical activity (days/week)			0.98 **	0.97–1.00	0.99 *	0.97–1.00
Walking (days/week)			0.98 ***	0.97-.99	0.99	0.98–1.00
Hypertension						
No			Ref.		Ref.	
Yes			1.06	1.00–1.13	1.02	0.95–1.08
Diabetes						
No			Ref.		Ref.	
Yes			1.26 ***	1.16–1.37	1.16 ***	1.07–1.27
Frequency of family and relatives contact						
≥4 times/week					Ref.	
1–3 times/week					1.03	1.00–1.11
1–3 times/month					1.01	0.93–1.09
None					1.11 *	1.01–1.22
Frequency of friend contact						
≥4 times/week					Ref.	
1–3 times/week					1.03	0.95–1.12
1–3 times/month					0.95	0.87–1.03
None					1.06	0.96–0.16
Frequency of neighbor contact						
≥4 times/week					Ref.	
1–3 times/week					0.87 **	0.81–0.95
1–3 times/month					0.87 **	0.79–0.95
None					1.04	0.96–1.12
Subjective stress						
Hardly					Ref.	
A little					1.24 ***	1.15–1.34
A lot					1.62 ***	1.48–1.77
Very much					1.96 ***	1.65–2.34
Depressive symptoms						
Minimal					Ref.	
Mild					2.71 ***	2.51–2.92
Moderate					4.28 ***	3.60–5.09
Moderately severe					4.85 ***	3.64–6.46
Severe					7.58 ***	5.05–11.38
Nagelkerke *R*^2^	0.02	0.03	0.09			
Wald *F/df*	48.86/12	40.24/20	63.82/36			

* *p* < 0.05, ** *p* < 0.01, *** *p* < 0.001. OR, odds ratio; 95% CI, 95% confidence interval; Ref., reference group.

**Table 4:** Factors influencing subjective cognitive impairment among older adults.

Variables	Model 1		Model 2		Model 3	
	OR	95% CI	OR	95% CI	OR	95% CI
Age	1.04 ***	1.04–1.05	1.04 ***	1.04–1.05	1.04 ***	1.04–1.05
Sex						
Male	Ref.		Ref.		Ref.	
Female	1.22 ***	1.17–1.28	1.43 ***	1.33–1.55	1.34 ***	1.24–1.44
Marital status						
Married	Ref.		Ref.		Ref.	
Single/others	1.12 *	1.02–1.23	1.11 *	1.01–1.22	1.14 *	1.03–1.25
Monthly income (USD)						
>5000	Ref.		Ref.		Ref.	
3001~5000	1.06	0.96–1.18	1.07	0.96–1.18	1.05	0.95–1.17
1001~3000	1.09 *	1.01–1.18	1.10 *	1.02–1.19	1.07	0.98–1.15
≤1000	1.21 ***	1.11–1.31	1.20 ***	1.11–1.30	1.06	0.98–1.15
Education level						
College or more	Ref.		Ref.		Ref.	
High school	1.05	0.96–1.16	1.04	0.95–1.15	1.03	0.93–1.13
Middle school	1.13 *	1.03–1.25	1.11 *	1.01–1.22	1.09	0.98–1.20
Elementary School or less	1.22 ***	1.12–1.34	1.19 ***	1.09–1.30	1.16 **	1.06–1.27
Living arrangement						
Living alone	Ref.		Ref.		Ref.	
Living with spouse	1.11	1.00–1.23	1.12 *	1.00–1.24	1.16 **	1.04–1.29
Living with parents or/and children	1.13 *	1.02–1.25	1.14 **	1.03–1.26	1.10	0.99–1.22
Others	1.07	0.98–1.17	1.08	0.99–1.18	1.03	0.94–1.12
Smoking						
No			Ref.		Ref.	
Yes			1.14 **	1.06–1.23	1.09 *	1.01–1.18
Alcohol drinking						
No			Ref.		Ref.	
Yes			1.24 ***	1.18–1.30	1.25 ***	1.18–1.31
Sleep (hours/day)			0.96 ***	0.94–0.97	1.01	1.00–1.03
Vigorous physical activity (days/week)			1.03 ***	1.01–1.05	1.04 ***	1.02–1.05
Moderate physical activity (days/week)			0.99 *	0.97–1.00	1.00	0.98–1.01
Walking (days/week)			0.97 ***	0.96–0.98	0.99	0.98–1.00
Hypertension						
No			Ref.		Ref.	
Yes			0.98	0.94–1.03	0.97	0.93–1.02
Diabetes						
No			Ref.		Ref.	
Yes			1.11 ***	1.06–1.17	1.06 *	1.01–1.12
Frequency of family and relatives contact						
≥4 times/week					Ref.	
1–3 times/week					1.11 **	1.04–1.17
1–3 times/month					1.15 ***	1.07–1.22
None					1.25 ***	1.16–1.34
Frequency of friend contact						
≥4 times/week					Ref.	
1–3 times/week					0.99	0.93–1.06
1–3 times/month					0.94	0.88–1.01
None					1.07	1.00–1.14
Frequency of neighbor contact						
≥4 times/week					Ref.	
1–3 times/week					1.05	0.99–1.12
1–3 times/month					1.09 *	1.01–1.18
None					1.12 ***	1.05–1.20
Subjective stress						
Hardly					Ref	
A little					1.16 ***	1.10–1.22
A lot					1.36 ***	1.26–1.47
Very much					1.75 ***	1.51–2.04
Depressive symptoms						
Minimal					Ref.	
Mild					2.34 ***	2.20–2.49
Moderate					3.31 ***	2.92–3.74
Moderately severe					4.26 ***	3.46–5.24
Severe					6.90 ***	5.02–9.50
Nagelkerke *R*^2^	0.04	0.04	0.11			
Wald *F/df*	78.78/12	58.07/20	76.05/36			

* *p* < 0.05, ** *p* < 0.01, *** *p* < 0.001. OR, odds ratio; 95% CI, 95% confidence interval; Ref., reference group.

## Data Availability

Publicly available data were used in this study. This data can be found here: https://chs.kdca.go.kr/chs/rdr/rdrInfoProcessMain.do (accessed on 24 March 2021).

## References

[1] Park B.H., Lee T.J., Lee Y.S., Jang S.H., Choi N.H., Han J.W., Yang H.J. (2019). Cost of illness and quality of life of patients and their caregivers with mild cognitive impairment or Alzheimer’s disease. J. Health Technol. Assess..

[2] Ko S.J., Jung Y.H., Kim D.Y. (2016). The Social Burden and Care Management for People with Dementia. Korean Institute for Health and Social Affairs Policy Report. Report No. 2016-04. https://www.kihasa.re.kr/publish/report/view?=research&seq=27752.

[3] Maresova P., Mohelska H., Dolejs J., Kuca K. (2015). Socio-economic aspects of Alzheimer’s disease. Curr. Alzheimer Res..

[4] National Institute of Dementia (2019). Korean Dementia Observatory. https://www.nid.or.kr/info/dataroom_view.aspx?bid=209.

[5] Gainotti G. (2010). Origins, controversies and recent developments of the MCI construct. Curr. Alzheimer Res..

[6] Peterson R.C. (2016). Mild cognitive impairment. Contin. Lifelong Learn. Neurol..

[7] Connors M.H., Quinto L., Brodaty H. (2019). Longitudinal outcome of patients with pseudodementia: A systematic review. Psychol. Med..

[8] Yoon K.A. (2017). Effects of caregiving burden on gain and family quality of life among dementia family caregivers: The moderating role of coping strategies. Korean J. Gerontol. Soc. Welf..

[9] Jessen F., Amariglio R.E., Vab Boxtel M., Breteler M., Ceccaldi M., Chetelat G., Dubois B., Dufouil C., Ellis K.A., van der Flier W.M. (2014). A conceptual framework for research on subjective cognitive decline in preclinical Alzheimer’s disease. Alzheimer’s Dement..

[10] Seo E.H. (2016). Pre-stage of mild cognitive impairment: Stage of subjective cognitive change. Korean J. Res. Gerontol..

[11] Center for Disease Control and Prevention (2018). Subjective Cognitive Decline-A Public Health Issue. https://www.cdc.gov/aging/data/subjective-cognitive-decline-brief.html.

[12] Kang Y.H., Hwang S.A., Park K.J. (2015). Reversion to normal cognition and its correlates among the community-dwelling elderly with mild cognitive impairment: The longitudinal cohort study. Korean J. Adult Nurs..

[13] Lee J.H., Sung J.Y., Choi M.K. (2020). The factors associated with subjective cognitive decline and cognitive function among older adults. J. Adv. Nurs..

[14] Zlatar Z., Muniz M., Galasko D., Salmon D.P. (2018). Subjective cognitive decline correlates with depression symptoms and not with concurrent objective cognition in a clinic-based sample of older adults. J. Gerontol. Psychol. Sci..

[15] Park H.J., Ha J.Y. (2020). Prediction models of mild cognitive impairment using the Korea longitudinal study of ageing. Korean J. Acad. Nurs..

[16] Park H.K., Song H.J. (2016). Predictors of cognitive function decline of elderly: Using living conditions and welfare needs of older Korean persons panel data. Korean J. Health Serv. Manag..

[17] Palta P., Carlson M.C., Crum R.M., Colantuoni E., Sharrett A.R., Yasar S., Nahin R.L., DeKosky S., Snitz B., Lopez O. (2018). Diabetes and cognitive decline in older adults: The ginkgo evaluation of memory study. J. Gerontol. Ser. A.

[18] Legdeur N., Heymans M.W., Comijs H.C., Huisman M., Maier A.B., Visser P.J. (2018). Age dependency of risk factors for cognitive decline. BMC Geriatr..

[19] Center for Disease Control and Prevention Behavioral Risk Factor Surveillance System. https://www.cdc.gov/brfss/.

[20] Oh J.Y., Yang Y.J., Kim B.S., Kang J.H. (2007). Validity and reliability of Korean version of international physical activity questionnaire. Korean J. Acad. Fam. Med..

[21] Kroenke K., Spitzer R.L., Williams J.B.W. (2010). The PHQ-9: Validity of a brief depression severity measure. J. Gen. Intern. Med..

[22] Luck T., Luppa M., Briel S., Matschinger H., König H., Bleich S., Villringer A., Angermeyer M.C., Riedel-Heller S.G. (2010). Mild cognitive impairment: Incidence and risk factors: Results of the leipzig longitudinal study of the aged. J. Am. Geriatr. Soc..

[23] Fonseca J.A.S., Ducksbury R., Rodda J., Whitfiels T., Nagaraj C., Suresh K., Stevens T., Walker Z. (2015). Factors that predict cognitive decline in patients with subjective cognitive impairment. Int. Psychogeriatr..

[24] Taylor C.A., Bouldin E.D., McGuire L.C. (2018). Subjective cognitive decline among adults aged ≥45 years—United States, 2015–2016. Morb. Mortal. Wkly. Rep..

[25] Hao L., Wang X., Zhang L., Xing Y., Guo Q., Hu X., Bin Mu M., Chen Y., Chen G., Cao J. (2017). Prevalance, risk factors, and complaints screening tool exploration of subjective cognitive decline in a large cohort of the Chinese population. J. Alzheimer’s Dis..

[26] Peterson R.L., Carvajal S.C., McGuire L.C., Fain M.J., Bell M.L. (2019). State inequality, socioeconomic position and subjective cognitive decline in the United States. SSM-Popul. Health.

[27] Mousavi-Nasab S.M.H., Kormi-Nouri R., Sundstrom A., Nilsson L. (2012). The effects of marital status on episodic and semantic memory in healthy middle-aged and old individuals. Scand. J. Psychol..

[28] Kim J.Y. (2017). Living arrangements and the distribution of multiple resources among Korean older adults. Korean J. Popul. Stud..

[29] Fondell E., Townsend M.K., Unger L.D., Okereke O.I., Grodstein F., Ascherio A., Willett W.C. (2018). Physical activity across adulthood and subjective cognitive function in older men. Eur. J. Epidemiol..

[30] Grande G., Vanacore N., Maggiore L., Cucumo V., Ghiretti R., Galimberti D., Scarpini E., Mariani C., Clerici F. (2014). Physical activity reduces the risk of dementia in mild cognitive impairment subjects: A cohort study. J. Alzheimer’s Dis..

[31] Hill N.L., Mogle J., Wion R., Munoz E., DePasquale M., Ycvchak A.M., Parisi J.M. (2016). Subjective cognitive impairment and affective symtopms: A systematic review. Gerontologist.

[32] Ahn S., Mathiason M.A., Yu F. (2021). Longitudinal cognitive profiles by anxiety and depressive symptoms in American older adults with subjective cognitive decline. J. Nurs. Scholarsh..

[33] Vlachos G.S., Cosentino S., Kosmidis M.H., Anastasiou C.A., Yannakoulia M., Dardiotis E., Hadjigeorgiou G., Sakka P., Ntanasi E., Scarmeas N. (2019). Prevalance and determinant of subjective cognitive decline in representative Greek elderly polulation. Int. J. Geriatr. Psychiatry.

[34] Podlesek A., Komidar L., Kavcic V. (2021). The relationship between perceived stress and subjective cognitive decline during the COVID-19 epidemic. Front. Psychol..

[35] Santos A.T.D., Leyendecker D.D., Costa A.L.S., Souza-Talarico J.N.D. (2012). Subjective memory complain in healthy elderly: Influence of depressive symptoms, perceived stress and self-esteem. Rev. Esc. Enfermagen USP.

[36] Littman A.J., White E., Satia J.A., Bowen D.J., Kristal A.R. (2007). Reliability and validity of 2 single-item measures of psychosocial stress. Epidemiology.

